# Enteral Feeding for Moderately Premature and Low Birth Weight Infants: A Single-Center Retrospective Observational Cohort Study

**DOI:** 10.1097/PG9.0000000000000288

**Published:** 2023-02-28

**Authors:** Dorita M.Z. Dekker, Monique van Brakel, Chris H.P. van den Akker, Frans B. Plötz

**Affiliations:** From the * Tergooi MC, Department of Pediatrics, Blaricum, The Netherlands; † Amsterdam UMC, location University of Amsterdam, Emma Children’s Hospital, Department of Pediatrics—Neonatology, Amsterdam, The Netherlands; ‡ Amsterdam Reproduction & Development Research Institute, Amsterdam, The Netherlands; § Amsterdam UMC, location University of Amsterdam, Emma Children’s Hospital, Department of Pediatrics, Amsterdam, The Netherlands.

**Keywords:** CPAP, feeding complications, newborns

## Abstract

Controversy exists in clinical practice regarding optimal initial enteral feeding (EF) for moderately premature and low birth weight (BW) infants. We included 96 infants stratified into 3 groups (I: 1600–1799 g [n = 22]; II: 1800–1999 g [n = 42]; III: 2000–2200 g [n = 32]). The protocol recommended starting with minimal EF (MEF) in infants weighing <1800 g. On the first day of life, 5% of the infants in group I did not follow the protocol mandating MEF, but started with exclusive EF instead, compared to 36% and 44% of the infants in groups II and III, respectively. The median number of days until exclusive EF was achieved was 5 days longer for infants receiving MEF than for infants who had received normal portions of EF from birth onward. We observed no significant differences in feeding-related complications. We advocate omitting MEF in moderately premature infants with a BW of 1600 g or higher.

**What Is Known**

-  Controversy exists in clinical practice regarding optimal initial enteral feeding (EF) for moderately premature and low birth weight (BW) infants.
-  The definition used for minimal EF is inconsistent and varies between 5 and 25 mL/kg/d.

**What Is New**

-  In healthy infants with a birth weight of 1600 g or higher we advocate to start with 20 mL/kg/d or higher EF from birth onwards.
-  To start with EF for infants while on continuous positive airway pressure support.

## INTRODUCTION

Controversy exists in clinical practice regarding initial optimal nutritional and total fluid management in preterm and low birth weight (BW) infants during the first week of life (1). This is particularly true for moderately preterm and low-birthweight infants who are often hospitalized in nonacademic hospitals. There are only a limited number of reports available on the evaluation of feeding protocols and their clinical compliance in this relatively large group of hospitalized patients (2,3). Different regimes are advocated, which include either starting with minimal EF (MEF) combined with parenteral nutrition (PN), enteral feeding (EF) in combination with intravenous glucose drip and/or PN, or exclusive EF thus without any intravenous fluids, directly from birth onwards. This controversy is further complicated by the definition used for MEF. These definitions are inconsistent and vary between 5 and 25 mL/kg/d (4–6).

To add to the knowledge bank, we herewith report our experiences in nutritional and fluid management for infants born with a BW between 1600 and 2200 g. The local protocol recommended starting with MEF in infants with a BW <1800 g and with slightly larger-sized portions of EF in infants with a BW >1800 g. For this study, we were interested in compliance with our feeding protocol, the time until exclusive EF, thus without any intravenous fluids, was met, and the frequency of feeding-related complications during clinical practice.

## METHODS

### Study Design

A single-center, retrospective, observational cohort study was conducted at Tergooi MC in Blaricum, Netherlands. This study was approved by the scientific review committee of Tergooi MC in May 2021 (study number: 21.26). This study was not subject to the Dutch Medical Research in Human Subjects Act.

### Participants

All infants with a BW between 1600 and 2200 g who were admitted to the neonatology ward of our hospital on the first day of life were included. In our hospital the neonatology ward is a 10-bed unit. Infants and mothers are hospitalized in separate locations. The included infants were stratified into three subgroups depending on BW: infants with a BW between 1600 and 1799, 1800 and 1999, and 2000 and 2200 g, respectively. The inclusion period was between January 2017 and March 2020 because an updated nutritional protocol was implemented in April 2020.

### Feeding and Total Fluid Protocol

The feeding and total fluid protocols during the first 8 days of life are presented in Supplemental Digital Table 1, http://links.lww.com/PG9/A99. It is noteworthy that every attending physician had the option to choose clinical management, not strictly according to the feeding protocol. Briefly, for infants with a BW <1800 g, the protocol recommended starting with MEF on the first day of life. MEF was defined as small portions of EF with a total volume of 15 mL/kg/d or less. The local feeding protocol recommended that EF be increased daily with small steps up to 12 times a day 14 mL on day 7, approximately 95–105 mL/kg/d. Total fluid requirements were achieved by providing intravenous glucose or full PN, which was increased stepwise during the first 3 days of life and decreased if, together with enteral nutrition, total fluid requirements were exceeded. PN was stopped if the EF exceeded 100 mL/kg/d.

For infants with a BW of 1800 g or higher, it was recommended to start after birth with normal portions of EF, defined as 8 times a day, 5–10 mL. The total fluid requirement was also achieved by providing additional intravenous glucose. EF was increased in steps to approximately 150 mL/kg/d on day 7 and the rates of intravenous fluids were decreased accordingly to attain total fluid requirements. However, in cases of severe dysmaturity (*z*-score for weight < −2.5 SD), sepsis, asphyxia, or if the infant received continuous positive airway pressure (CPAP) respiratory support, only MEF was started after birth in these infants in combination with PN and/or glucose drip, possibly continued for several days.

### Data Collection and Definitions

Information about sex, BW, gestational age, small for gestational age (SGA) incidence, birth mode, Apgar scores, and maternal incidence of diabetes gravidarum were collected. For the first 7 days of life, the need for respiratory CPAP support and antibiotic therapy for (suspected) infection were recorded. Data on EF-related disorders including gastric residuals, vomiting, and necrotizing enterocolitis were collected. Finally, data on the number of central venous line placements for PN administration were obtained. The data were entered in castor electronic data management system (EDS).

SGA was defined as a BW below the 10th percentile for matching gestational age according to the specified growth curve. Exclusive EF was defined as an EF without intravenous fluid administration.

### Statistical Analysis

The Statistical Program of the Social Sciences (SPSS; version 26) was used for analysis. Statistical analyses were performed using the Kruskal–Wallis test. Statistical significance was set at *P* <0.05.

## RESULTS

### Participants

During the study period, 132 infants admitted to our neonatal high care ward had a BW between 1600 and 2200 g. We excluded 36 infants, of whom 16 were not born at Tergooi MC and 20 not admitted on the first day of life but thereafter from the maternity ward. The remaining 96 infants were included and stratified into three groups: 22 infants with a BW between 1600 and 1799 g, 42 infants between 1800 and 1999 g, and 32 infants between 2000 and 2200 g. Demographic data and number of infants who received CPAP and antibiotic therapy are presented in Table 1.

**TABLE 1.** Baseline characteristics.

	BW 1600–1799 g (n = 22)	BW 1800–1999 g (n = 42)	BW 2000–2200 g (n = 32)	Total (n = 96)
Gender, male (%)	10 (45)	21 (50)	15 (47)	46 (48)
Birth weight (mean [g], SD)	1707 (56)	1910 (64)	2118 (60)	1933 (165)
Gestational age (mean [weeks], SD [days])	34.6 (12)	34.3 (11)	34.7 (7)	34.4 (10)
Small for gestational age (%)*	11 (50)	14 (33)	6 (19)	31 (32)
Vaginal delivery (N, %)	9 (41)	30 (71)	20 (63)	59 (61)
Apgar score 5 min (median, range)	10 (8–10)	10 (5–10)	10 (6–10)	10 (5–10)
Apgar score 10 min (median, range)	10 (9–10)	10 (7–10)	10 (8–10)	10 (7–10)
Diabetes gravidarum (N, %)	1 (5)	3 (7)	3 (9)	7 (7)
Number of infants on CPAP (N, %)	5 (23)	10 (24)	13 (41)	28 (29)
Number of days of CPAP (median, range)	2 (1–4)	1 (1–4)	1 (1–3)	1 (1–4)
Number of infants who received antibiotic therapy after birth (N, %)	10 (45)	16 (38)	12 (38)	38 (40)
Number of infants who received antibiotic therapy less than 72 hours (N, %)	3 (14)	10 (24)	8 (25)	21 (22)

CPAP = continuous positive pressure ventilation.

*SGA was defined as a BW below the 10th percentile for the matching gestational age.

### Outcomes

#### Start of EF

On the first day of life, one infant (5%) with a BW between 1600 and 1799 g started with exclusive EF, despite the local feeding protocol mandating only MEF together with intravenous fluids. In the higher-weight groups, exclusive EF from birth onwards was achieved in larger proportions: 36% of the infants with a BW between 1800 and 1999 g and 44% of the infants with a BW between 2000 and 2200 g (Table 2). Thus, these infants did not receive additional glucose drips and/or PN to meet their fluid requirements. On the seventh day of life, the percentage of exclusive EF increased to 45%, 74%, and 81%, respectively. Infants with a BW between 1800 and 2200 g who had received MEF were all on CPAP support.

**TABLE 2.** The distribution of MEF, EF combined with a GD and/or parenteral nutrition (PN), and exclusive EF in the 3 different birth weight categories on the first and seventh day of life

	1600–1799 g		1800–1999 g		2000–2200 g	
	Day 1, N = 22	Day 7, N = 22	Day 1, N = 42	Day 7, N = 42	Day 1, N = 32	Day 7, N = 32
MEF + GD and/or PN	17 (77%)	1 (5%)	7 (17%)	0 (0%)	7 (22%)	0 (0%)
EF + GD and/or PN	4 (18%)	11 (50%)	20 (48%)	11 (26%)	11 (34%)	6 (19%)
Exclusive EF	1 (5%)	10 (45%)	15 (36%)	31 (74%)	14 (44%)	26 (81%)

EF = enteral feeding; GD = glucose drip; MEF = minimal enteral feeding; PN = parenteral nutrition.

#### Number of Days Until Exclusive EF

The median number of days until exclusive EF, thus without any intravenous fluid administration, was reached in infants in the 1600–1799 g group amounted to 8 days (range 1–14) (Table 3). For infants with a BW between 1800 and 1999 g, this amounted to 4.5 days (range 1–16), and in the 2000–2200 g group, it was 4 days (range 1–10). For infants with a BW between 1800 and 1999 g, who started EF during the first 7 days of life, it was 4 days (range 1–11) compared to 9 days (range 4–16) for infants who started with MEF.

**TABLE 3.** Median number of days until exclusive enteral feeding was reached within the birth weight subgroups.

	1600–1799 g		1800–1999 g		2000–2200 g			
	N	Median (range)	N	Median (range)	N	Median (range)	H	*P*
All infants	22	8.0 (1–14)	42	4.5 (1–16)	32	4.0 (1–10)	11.065	0.004‡
Minimal enteral feeding*	17	8.0 (5–14)	7	9.0 (8–16)	7	5.5 (4–8)		
Enteral feeding†	5	5.0 (1–6)	35	4.0 (1–11)	26	3.0 (1–10)		

H = Kruskal–Wallis H statistic.

*Infants who started with minimal enteral feeding on one or multiple days.

†Infants who received normal portions of enteral feeding all 7 days.

‡Kruskal–Wallis test was run to asses if there were differences in median days until full enteral feeding between the 3 subgroups.

#### Complications of Feeding

We found no differences in the occurrence of feeding-related complications among the three groups. Necrotizing enterocolitis did not occur in any of the infants. The requirement for a central venous line to administer PN occurred only in one infant in the 1600–1799 g group.

## DISCUSSION

This study evaluated during clinical practice nutritional and fluid management during the first seven days of life in moderately premature or low-BW infants hospitalized in a level 2 neonatal ward in a teaching hospital. We observed that the percentage of infants who started immediately after birth with exclusive EF, without any additional intravenous glucose or PN, increased with higher BW. In turn, providing only MEF after birth also resulted in a longer median duration of achieving exclusive EF compared with infants who received slightly larger portions of EF from birth onwards. The occurrence of feeding-related complications did not differ between infants who started after birth with MEF and those who started with slightly larger portions of EF.

Initially, this feeding and fluid management protocol was established for all premature infants with a gestational age between 32 and 36 weeks and a BW between 1200 and1800 g. For these infants, the protocol recommends starting MEF combined with PN to achieve fluid requirements. The lower BW limit was based on the fact that infants in our hospital were only admitted with a minimal BW of 1200 g, whereas an upper BW limit of 1800 g was arbitrarily chosen. The results of our study, however, show that for infants with a BW between 1600 and 1800 g, this is a very conservative approach. If this protocol is strictly applied, these infants are still in need of PN and/or intravenous glucose 7 days after birth to meet their fluid requirements. Consequently, infants are frequently exposed to achieve vascular access, and may eventually require a central venous line, which is the case in a single infant. In clinical practice, adherence to the protocol was moderate. Although the majority of infants in the different BW groups started with MEF or EF according to the protocol, not all infants received additional PN and/or intravenous glucose to meet their fluid requirements. One reason could be that several attending physicians found the protocol to be too conservative.

To ensure uniform clinical practice, we advocate abandoning only MEF after birth in these patient groups but start with slightly larger portions of EF on the first day of life. This is in line with the results of a randomized controlled trial, in which SGA infants of gestational age 32–36 weeks and BW >1499 g were randomly allocated to receive either a proactive feeding regime, starting with 100 mL/kg/d and gradually increasing to 200 mL/kg/d by day 4, or a standard feeding regimen, starting at 60 mL/kg/d and gradually increasing to 170 mL/kg/d by day 9 (7). All the infants received human milk. They found that a proactive feeding regimen in moderately preterm SGA infants was well-tolerated. Infants assigned to the proactive feeding regimen received significantly less intravenous fluids, had less weight loss, and a significantly faster regain of BW, whereas feeding intolerance did not differ when compared to the controls. The incidence of neonatal hypoglycemia and length of stay were also significantly reduced. It must be noted that even the conservative approach in this trial is much more rapid in advancing EF than in our cohort.

Another conservative approach in our protocol is to start with MEF when CPAP is applied. However, since recent experiences and literature suggest that even the controlled introduction of oral bottle feeding for infants on CPAP can be safe, adjustments to our protocol are justified to start with and increase nasogastric tube feeding more rapidly than providing only MEF for infants while on CPAP support (8).

A major strength of our cohort is that this is a study that reports compliance to and suggestions on how to improve a feeding protocol for these groups of infants. Evidence-based nutritional guidelines for moderately preterm infants and low-BW infants are notably absent, despite being the largest population of preterm infants. A recent review confirmed this (9). The authors described the nature and evidence base of internationally available guidelines for the introduction of oral feeding in preterm infants in neonatal units. They found great variations in their recommendations and details of interventions, and advocated the development of evidence-based guidelines.^9^ In addition, to assess guidelines in clinical practice we also advocate to develop a core outcome set. Key outcomes that might be included could be avoidance of hypoglycemia, time to feeds, breastfeeding success, or growth. This will also increase comparison between studies and improve the management of these infants.

Our study is a retrospective cohort that is always dependent on accurate record keeping, and is therefore a limitation. However, our study mostly covers requested feeding and intravenous glucose and PN parameters, which were recorded digitally in electronic patient files, and therefore, most likely none of these parameters could be missed. Another limitation is that our feeding protocol was only loosely followed; therefore, it was difficult to interpret the clinical tolerance of our feeding protocol in each weight class. Finally, it is a single-center study and this limits the generalizability of this study.

In conclusion, based on this observational study and the existing literature, we recommend starting with 20 mL/kg/d or higher EF from birth onward in moderately premature healthy infants with a BW of 1600 g or higher. Furthermore, we recommend the development of international evidence-based guidelines for this group of moderately premature and low-BW infants and to develop a set of core outcome parameters. This may help achieve uniform clinical practice as well to assess the guidelines in clinical practice.

## Supplementary Material

## References

[1] Lapillonne A Bronsky J Campoy C. Feeding the late and moderately preterm infant: a position paper of the European Society for Paediatric Gastroenterology, Hepatology and Nutrition Committee on Nutrition. J Pediatr Gastroenterol Nutriton. 2019;69:259–70. 10.1097/MPG.0000000000002397 31095091

[2] Baillat M Pauly V Dagau G. Association of first-week nutrient intake and extrauterine growth restriction in moderately preterm infants: a regional population-based study. Nutrients. 2021;13:227. 33466801 10.3390/nu13010227 PMC7830065

[3] Gerritsen L Lindeboom R Hummel T. Prescribed protein intake does not meet recommended intake in moderate- and late-preterm infants: contribution to weight gain and head growth. Nutr Clin Pract. 2020;35:729–37. 32125013 10.1002/ncp.10464

[4] Salas AA Kabani N Travers CP. Short versus extended duration of trophic feeding to reduce time to achieve full enteral feeding in extremely preterm infants: an observational study. Neonatology. 2017;112:211–6. 28704816 10.1159/000472247

[5] Dutta S Singh B Chessell L. Guidelines for feeding very low birth weight infants. Nutrients. 2015;7:423–42. 25580815 10.3390/nu7010423 PMC4303848

[6] Morgan J Bombell S McGuire W. Early trophic feeding versus enteral fasting for very preterm or very low birth weight infants. Cochrane Database Syst Rev. 2013;28:CD000504. 10.1002/14651858.CD000504.pub4 PMC11480887 23543508

[7] Zecca E Costa S Barone G. Proactive enteral nutrition in moderately preterm small for gestational age infants: a randomized controlled trial. J Pediatr. 2014;165:1135–1139.e1. 25304922 10.1016/j.jpeds.2014.08.065

[8] Shetty S Hunt K Douthwaite A. High-flow nasal cannula oxygen and nasal continuous positive airway pressure and full oral feeding in infants with bronchopulmonary dysplasia. Arch Dis Child Fetal Neonatal Ed. 2016;101:F408–411. 26883075 10.1136/archdischild-2015-309683

[9] Bakker L Jackson B Miles A. Oral-feeding guidelines for preterm neonates in the NICU: a scoping review. J Perinatol. 2011;41:140–9. 10.1038/s41372-020-00887-6 33288867

