# Supplementary material for: Enteral Feeding for Moderately Premature and Low Birth Weight Infants: A Single-Center Retrospective Observational Cohort Study
Source: JPGN Rep. 2023 Feb 28;4(2):e288. doi: 10.1097/PG9.0000000000000288 (PMC10187862; doi:10.1097/PG9.0000000000000288)
Supplement: Supplementary file 1 [file pg9-4-e288-s001.pdf]

Supplemental table 1. Daily feeding schedule and total fluid recommendations for infants with a birthweight of 1200-1800 gram and infants with a birthweight of >1800 gram according to the protocol 'Nutrition for Infants' of the Tergooi MC.

	1200-1800 gram		>1800 gram*	
Day	*Standard feeding schedule***	*Total fluid****	*Standard feeding schedule***	*Total fluid****
1	6x2 mL or 12x2 mL	60 mL/kg/day	8x5-10 mL	40 mL/kg/day
2	12x3 mL	80 mL/kg/day	8x10-15 mL	60 mL/kg/day
3	12x6 mL	100 mL/kg/day	8x20 mL	80 mL/kg/day
4	12x8 mL	120 mL/kg/day	8x30 mL	100 mL/kg/day
5	12x10 mL	120-140 mL/kg/day	8x40 mL	120 mL/kg/day
6	12x12 mL	140-160 mL/kg/day	8x50 mL	140 mL/kg/day
7	12x14 mL	140-160 mL/kg/day	8x 50 mL	150 mL/kg/day
8	12x16 mL	160 mL/kg/day	7x60 mL	160-180 mL /kg/day

* *In this weight class MEF is advised in case of very preterm birth (<32 weeks), severe growth restriction (<-2.5 SDS), perinatal asphyxia, the requirement of continuous positive airway pressure (CPAP), or suffering sepsis.*

** *Recommended portions of enteral feeding.*

*** *Total fluid consist of enteral feeding supplemented by glucose drip and/or total parenteral nutrition.*
